# Computational extraction of a neural molecular network through alternative splicing

**DOI:** 10.1186/1756-0500-7-934

**Published:** 2014-12-19

**Authors:** Shafiul Alam, Huong Thi Thanh Phan, Mio Okazaki, Masahiro Takagi, Kozo Kawahara, Toshifumi Tsukahara, Hitoshi Suzuki

**Affiliations:** School of Materials Science, Japan Advanced Institute of Science and Technology, Nomi, Ishikawa, 923-1292 Japan; Department of Chemicals and Engineering, Miyakonojo National College of Technology, Miyakonojo, Miyazaki, 885-0006 Japan; World Fusion Co., Ltd, Chuo-ku, Tokyo, 103-0013 Japan; Center for Nano Materials and Technology, Japan Advanced Institute of Science and Technology, Nomi, Ishikawa, 923-1292 Japan

**Keywords:** Comprehensive analysis, Neuronal differentiation, Alternative splicing

## Abstract

**Background:**

Generally, the results of high throughput analyses contain information about gene expressions, and about exon expressions. Approximately 90% of primary protein-coding transcripts undergo alternative splicing in mammals. However, changes induced by alternative exons have not been properly analyzed for their impact on important molecular networks or their biological events. Even when alternative exons are identified, they are usually subjected to bioinformatics analysis in the same way as the gene ignoring the possibility of functionality change because of the alteration of domain caused by alternative exon. Here, we reveal an effective computational approach to explore an important molecular network based on potential changes of functionality induced by alternative exons obtained from our comprehensive analysis of neuronal cell differentiation.

**Results:**

From our previously identified 262 differentially alternatively spliced exons during neuronal cell differentiations, we extracted 241 sets that changed the amino acid sequences between the alternatively spliced sequences. Conserved domain searches indicated that annotated domain(s) were changed in 128 sets. We obtained 49 genes whose terms overlapped between domain description and gene annotation. Thus, these 49 genes have alternatively differentially spliced in exons that affect their main functions. We performed pathway analysis using these 49 genes and identified the EGFR (epidermal growth factor receptor) and mTOR (mammalian target of rapamycin) signaling pathway as being involved frequently. Recent studies reported that the mTOR pathway is associated with neuronal cell differentiation, vindicating that our approach extracted an important molecular network successfully.

**Conclusions:**

Effective informatics approaches for exons should be more complex than those for genes, because changes in alternative exons affect protein functions via alterations of amino acid sequences and functional domains. Our method extracted alterations of functional domains and identified key alternative splicing events. We identified the EGFR and mTOR signaling pathway as the most affected pathway. The mTOR pathway is important for neuronal differentiation, suggesting that this *in silico* extraction of alternative splicing networks is useful. This preliminary analysis indicated that automated analysis of the effects of alternative splicing would provide a rich source of biologically relevant information.

**Electronic supplementary material:**

The online version of this article (doi:10.1186/1756-0500-7-934) contains supplementary material, which is available to authorized users.

## Background

Approximately 23,000 human protein coding genes have been identified; however, this is a much smaller number than the expected over 200,000 human proteins [1]. Alternative splicing changes the use of exons, producing multiple transcripts from a single gene, and enhances proteomic diversity to support complexity in higher eukaryotes [2]. Indeed, it was reported that approximately 90% of human genes undergo alternative splicing [3]. There are five basic models of alternative splicing: exon skipping, mutually exclusive type, 5’ splice site selection, 3’ splice site selection, and intron retention. Additionally, alternative promoters and alternative polyadenylation sites can produce alternative isoforms of transcripts. These exons are sometimes continuously located on the genome, resulting in complex alternative splicing [4].

According to physiological or environmental changes, some alternative splicing occurs in a spatiotemporal manner, regulated by alternative splicing regulators. Several regulators, such as Nova1, RBFox1 and nPTB, and their targets, have been identified in neural tissues and cells [5–7]. Many isoforms produced by alternative splicing have distinctly different functions, and play important biological roles [8, 9]. Therefore, it is important to determine critical alternative splicing networks based on biological phenomena.

Over the last decade, many high throughput analyses have been performed, and their information has accumulated in databases. Both gene level expression and exon level expression are available from analyses using large-scale sequencing technologies. Furthermore, recently developed standard microarrays may provide information on both gene and exon expressions [10]. Certain differentially expressed genes significantly affect biological phenomena or are useful as molecular markers; therefore, information on gene level expressions is generally well analyzed. Moreover, critical gene networks or target genes can be identified from gene expression information. However, this is not the case for exons.

Complicated species of alternative isoforms with small numbers of nucleotide changes can be predicted compared with genes. Thus, the quantitative credibility of an exon’s information is generally much less than that of genes. Moreover, the analysis of exons is more complex than that of genes. Although some differentially alternatively spliced exons have been investigated and extracted, the annotated genes of these exons may be used to examine the critical networks or targets [11–13]. However, significant changes resulting from alternative exons may occur in protein domain(s). Advanced molecular dynamics techniques were also applied to investigate alternative isoforms of particular individual isoforms [14–18]. These methods are not currently applicable to the genome-wide investigation of alternative isoforms at a time. Of course, several specialized programs support informatics analyses of alternative exons [19–22]. However, these do not support analyses of the protein domains encoded on alternative exons. In the case of individual small-scale analysis, researchers generally check alterations of amino acid sequences and of functional protein domains according to usages of alternative exons. AltAnalyze and DomainGraph could provide protein domain information from alternative exons although this type of analysis of exons has been rarely applied comprehensively [23].

Previously, we analyzed comprehensively the differentially alternatively spliced exons during neuronal differentiation of P19 mouse embryonic carcinoma cells [12]. Validation by reverse-transcription polymerase chain reaction (RT-PCR) suggested that 87% of the obtained 262 exons were differentially alternatively spliced in neuronal cells compared with undifferentiated cells. Moreover, many of the genes that were annotated by 262 exons were suggested to be involved in neural events. Thus, the 262 exons were plausible as neural splicing exons, and we considered that these exons could be a good example group to investigate alterative splicing networks.

In this article, we searched for a network that involved these alternative splicing events using functional domain information. Although individual studies have focused on the functionalities of alternative spliced domains, all comprehensively obtained exons were used as objects in the domain functionality search. Ultimately, we identified the EGFR (epidermal growth factor receptor) and mTOR (mammalian target of rapamycin) signaling pathway as the molecular network most associated with neural alternative splicing. The EGFR/mTOR pathway is an important cellular signaling pathway that controls cell growth and proliferation [16, 24]. This pathway has been intensively studied, and many of the proteins involved have been identified. Recently, studies have found that the mTOR pathway plays important roles in the maintenance of neural stem cells and the differentiation of neuronal cells [reviewed in [25]. However, the relationship between the EGFR/mTOR pathway and alternative splicing remains unknown. Our investigation suggested that gene expression and exon expression of the transcripts involved in this pathway were dramatically changed during neural differentiation of P19 cells. Indeed, our trial showed that searching for molecular network according to the functionalities of alternatively spliced domains was an effective strategy. Thus, it will help to precisely analyze mega data that are available from comprehensive analyses.

## Methods

### Data set of the exon array during neuronal differentiation of P19 cells

The accession number of the exon Array data is GSE23710, which we analyzed and reported previously [12]. Information on the DAS exons, such as probeset sequences, is available in the supplemental table of that report [12], as is the information on the differentially expressed genes.

### Collection of annotated sequence data from the probeset sequences

All probeset sequences were analyzed in the UCSC Blast-like alignment (blat) search tool (http://genome.ucsc.edu/cgi-bin/hgBlat) [26], and we determined alternative exons or region sequences compared with the annotated Refseq sequence, mRNA sequence or EST sequences. Similarly, we manually selected the most typical and representative sequences in which the alternative exon (or region) was either joined to or excised from a transcript. All transcript sequences were translated into amino acid sequences using the ExPASy translate tool (Swiss Institute of Bioinformatics). Sequences of the determined DAS exons were also translated into amino acid sequences. Whole amino acid sequences, excluding or including alternative exons or regions, were compared and validated in the UCSC blat search.

### Conserved domain search for alternatively spliced isoforms

The obtained amino acid sequences were analyzed in the NCBI conserved domain search [27], and alternatively spliced domains were determined. Additionally, text descriptions of these domains were retrieved.

### Gene ontology analysis, pathway analysis and text mining

The GO analyses were performed for 128 DAS genes with altered domain(s) [28]. The GO term(s) were compared with the text descriptions of the domain(s). Overlaps between GO terms and domain descriptions were found in 49 out of 128 genes. These 49 genes were subjected to pathway analysis and statistically-significant (Fisher’s Exact Test *p* ≤ 0.01) biological process terms were obtained using PathwayStudio® (Ariadne Genomics Inc., Rockville, MD, USA) [28, 29]. Although the schematic representation of pathway analysis was based on this analysis, their relationships were validated by KEGG pathway analysis (http://www.genome.jp/kegg/pathway.html) and by previous articles for Arap1, Ep400 and Arhgef12 [30–32].

### Cell culture and RNA purification

P19 cells were maintained in α minimum essential medium (α-MEM; Sigma–Aldrich, St. Louis, MO, USA) supplemented with 10% fetal bovine serum (FBS; Sigma–Aldrich) [33]. To induce neuronal cell differentiation, P19 cells (1 × 10^5^ cells/mL) were treated with 1 μM all trans-retinoic acid at 4°C in a 10 cm petri dish (Falcon) with α-MEM containing 10% FBS, as described previously [33, 34]. Total RNAs were collected from undifferentiated cells (Day 0), cells during neuronal induction (Day 1–4), differentiation (Day 5–9), and from cells in the early glial stage (Day 10–12) using the Trizol reagent (Life Technologies, Grand Island, NY, USA).

### Semi-quantitative RT-PCR

The total RNAs of P19 cells were prepared as described above. The cDNAs were produced from 2 μg of total RNAs using SuperScript III (Life technologies) and 0.5 μg of oligo dT primer in a 20 μL reaction mixture. The cDNAs are used as templates for PCR. GoTaq polymerase (Promega, Fitchburg, WI, USA) with specific primers performed the PCR reactions. The DNA primer sequences, number of cycles and annealing temperatures for the candidates are described in Additional file 1: Table S1. Primers and conditions for *β-Actin* and *GluR1* (controls) were described in a previous report [34]. The PCR products were analyzed on a 6% polyacrylamide gel. The gels were stained with SYBR Green I (Takara Bio Inc., Otsu, Japan), and a LAS-3000 (GE Healthcare, Fairfield, CT, USA) was used to analyze the images. The sequences of the PCR products were confirmed in a 3100 DNA sequencer (Life technologies). MultiGauge v 3.0 software (GE Healthcare) was used to perform the densitometry. Each experiment was performed at least three times to confirm reproducibility.

As for Ethics, this research did not involve any human subject, human material, or human data, and was not performed on any animals. This research involved Recombinant DNA Experiments and was approved by Life science committee of Japan Advanced Institute of Science and Technology.

## Results and discussion

### Extraction of important genes regulated by alternative splicing

**Figure 1:**
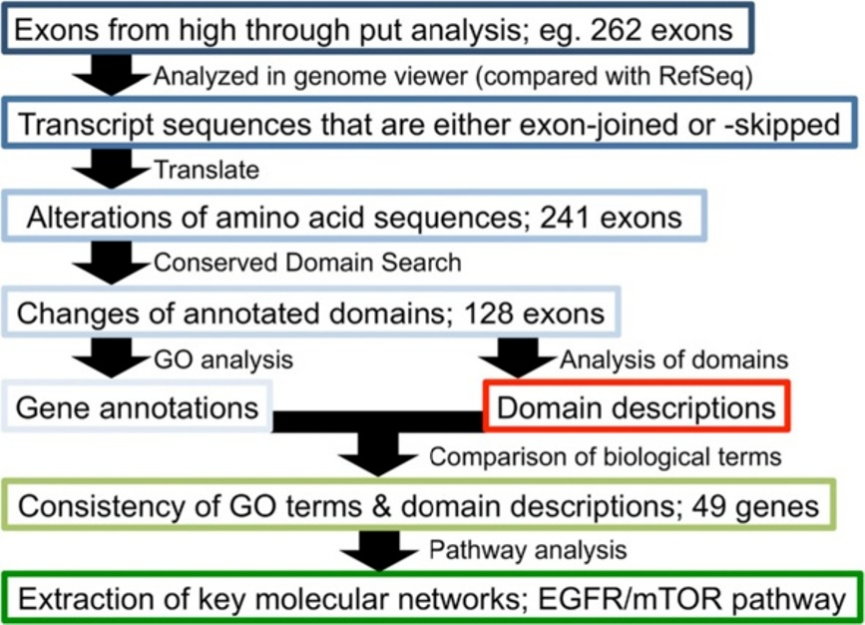
**Flow chart of the comprehensive informatics approach to alternative splicing.** 262 differentially alternatively spliced (DAS) exons obtained by Exon Array analysis were tested because it was suggested that approximately 87% of these DAS exons were changed during neuronal differentiation of P19 cells [12]. The exons were determined and their transcripts with/without DAS exons were collected and translated. A conserved domain search suggested that the functional domains could be changed in 128 pairs via alternative splicing. Moreover, comparison of gene annotations and their domain descriptions suggested that alternative splicing in 49 genes affected their critical functional domain. Pathway analysis suggested that the EGFR/mTOR signaling pathway could be the most affected molecular network.

**Figure 2:**
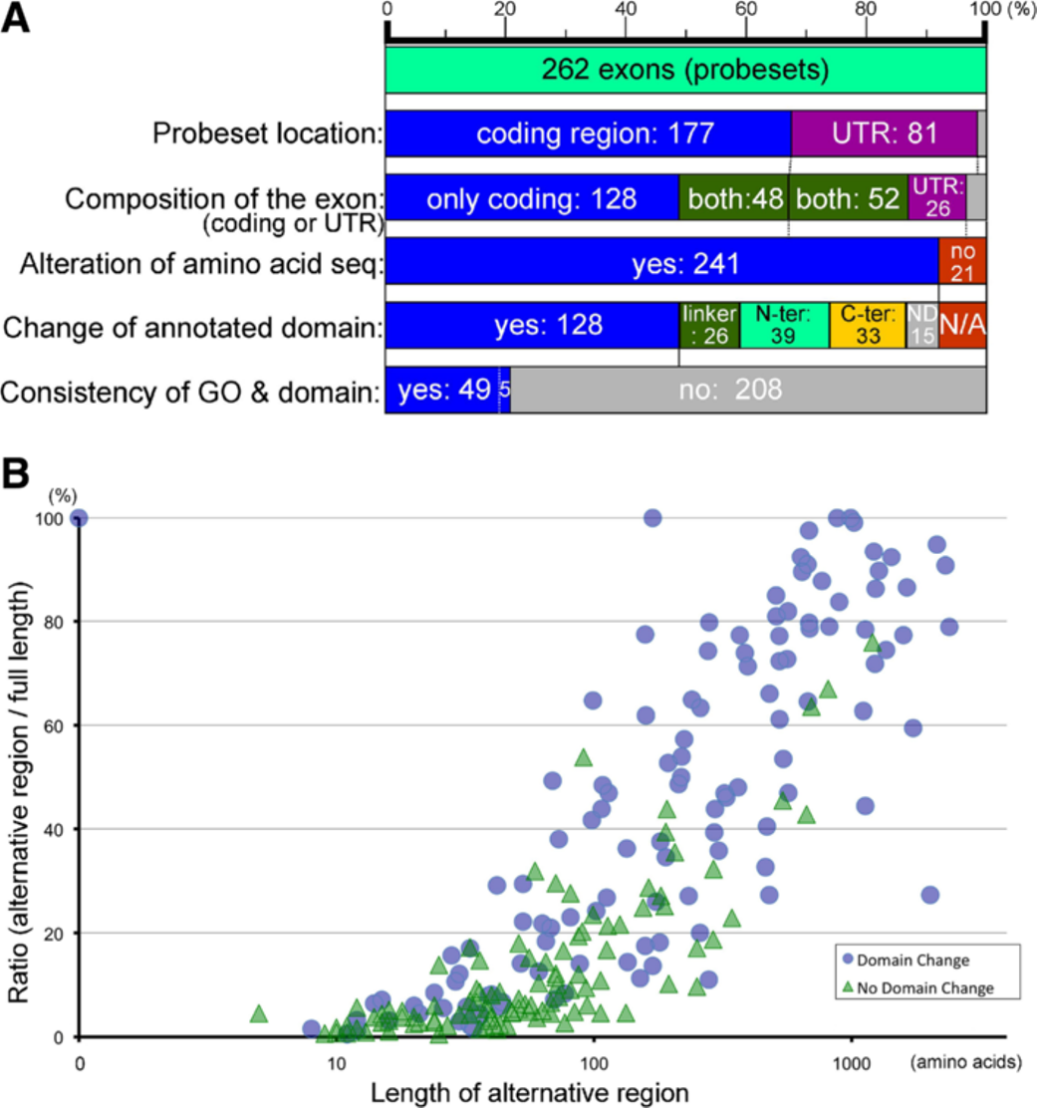
**Graphical representations of the relationship of probesets sequences to protein domains. (A)** Comprehensively obtained signals were originally annotated as probeset sequences. All probeset sequences of the 262 DAS exons were manually examined to determine whether these sequences were protein coding regions or untranslated regions (UTR). Each alternative exon sequence was determined based on the annotated Refseq, mRNAs and ESTs in genome viewer. Among the 262 determined exon sequences, approximately half of them are protein-coding exons (3rd bar, blue). Approximately 40% of the exons sequences contained both protein coding region and UTR (3rd bar, green). Only 26 exons comprised UTR without coding region (3rd bar, violet). Ultimately, 241 DAS exons were predicted to affect amino acid sequences (protein sequences). Moreover, 128 DAS exons were predicted to affect protein domain(s). Finally, 49 genes (54 probeset seqs, including five probeset seqs that indicated the same exons and/or domains) were extracted whose representative function was predicted to be affected by alternative splicing. Grey color indicates the not-determined exons. **(B)** The relationship between length of the altered protein sequences and domains are shown. A dot indicates an exon that changed a protein domain(s). A triangle indicates an exon that did not change a domain. A cross indicates an exon that did not change the protein sequence. DAS exons that are regulated by alternative promoters or polyadenylation may affect longer amino acid sequences with a high occupancy rate, and are likely to more frequently affect protein domain(s), as observed in upper-right area of the panel. Meanwhile, it is predicted that some short alternative exons also are able to change protein domains.

Previously, we obtained 262 candidate exons (236 genes) that were differentially alternatively spliced during neuronal differentiation of P19 cells [12]. Validation by RT-PCR indicated that the expressions of 87% of them actually changed; therefore, we used the 262 exons as good example for subsequent analysis (Figure 1). This was performed in a mouse Exon Array; therefore, intact information of these exons was provided as probeset sequences (probeset seqs). These probeset seqs were analyzed in a genome viewer; human blat analysis. According to annotated Refseqs, mRNAs or ESTs, the 262 alternative exons were analyzed and the two entire amino acid sequences that the alternative exon candidate joined or excised in its transcripts were determined (Figure 1, Additional file 2: Table S2). Occasionally, several Refseqs were annotated at the genome locus. In these cases, we selected the most plausible and representative transcript that involved in the alternative exon. Although 81 probeset seqs were located in the untranslated regions (UTRs), 52 probeset seqs indicated exons that contained protein-coding sequences (Figure 2A). Moreover, some alternative terminations by non-coding UTR exons could cause changes in the amino acid sequence. Altogether, 241 entire transcript sequences producing changes of amino acid sequence by including and excluding the alternative exon were identified (Figures 1 and 2A, Additional file 2: Table S2).To clarify the effects on functional domains of the alternative exons, we performed a conserved domain search at NCBI. Among 241 variable coding regions, 128 might cause changes to the annotated domains (Figures 1 and 2A). The others were suggested to affect N-terminal regions, C-terminal regions and linker region (inter-domains) (Figure 2A). In the case of alternative promoters or polyadenylation, their upstream or downstream exons could be excluded from its transcript, respectively. Therefore, some alternative exons are responsible for longer amino acid sequences and affect large parts of the amino acid sequence. These case are shown in the upper right area of Figure 2B and these drastic changes of amino acid sequences mostly caused functional domain changes (blue dots). In addition, many alternative splicings changed < 100 amino acids and < 20% of the full-length protein, as shown in lower left area of Figure 2B. Some of them also produced alterations in functional protein domains (Figure 2B).For the 128 regions that changing their domains, we extracted the descriptions of their domains from the NCBI conserved domain search. We also performed Gene Ontology (GO) analysis of the corresponding 128 genes. We compared the domain descriptions with the GO terms, and found 49 genes (54 probeset seqs, including five probeset seqs that indicated the same exons and/or domains) that had words in common, not including conjunctions and prepositions (Figures 1 and 2A). Thus, the critically important molecular functions of 49 genes could be affected or regulated by their differentially alternatively spliced exons.

### Pathway analysis based on the domain change

**Figure 3:**
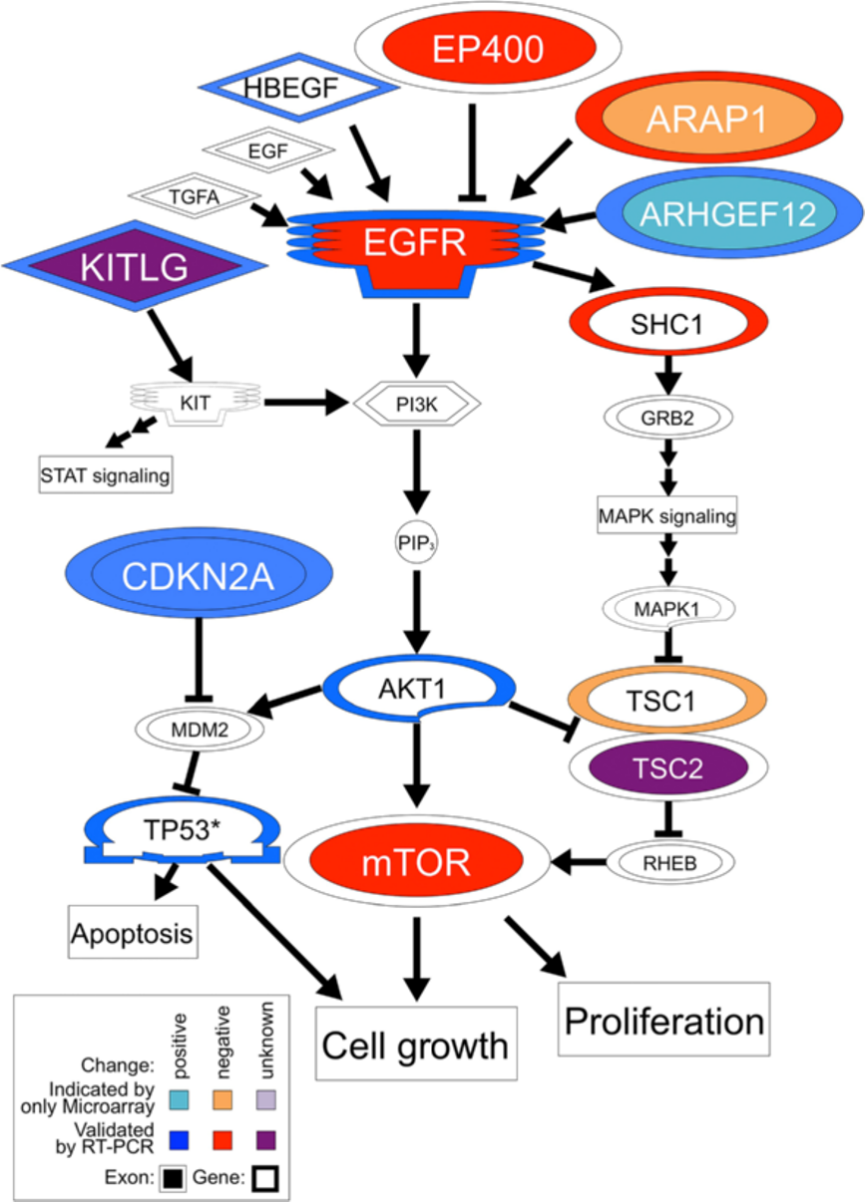
**Pathway analysis of 49 genes whose functional domains were changed by DAS exons.** The EGFR/mTOR signaling pathway was analyzed using 49 genes, where the functionalities of the changed domains were related to gene annotations. Thus, representative functions of these genes may be regulated by alternative splicing events. Seven of them were shown with larger fonts with an inner color. The lines indicate the intermolecular relationships. Medium fonts indicate differentially expressed genes suggested by a previous analysis [12]. Outer colors indicate the elevation or reduction of their expressions. Smaller fonts indicate closely related upstream or downstream proteins. Blue or red indicate successfully validated gain of function and elevation of expression or loss of function and reduction of expression, respectively. Success of the validation by semi-quantitative RT-PCR was determined by an Fc value > 1.5 between undifferentiated P19 cells (day 0) and the cells at the neuronal stage (day 7) (see also Figure 4). Light blue or orange colors indicate unsuccessful validation, even though these genes were suggested by a previous microarray experiment. The violet inner color of Kitlg indicates that its domains were suggested to change but the gain or loss of function was difficult to determine. Tsc2 was not one of the extracted 49 genes, but was one of 262 DAS exon genes. Indeed, its alternative isoforms have no difference in their functional protein domains. Additionally, the transcript of p53 (TP53) was not listed as a differentially expressed gene; however, its protein level was likely to be upregulated, as described in a previous report [37].

To find molecular networks or pathways that involved the differentially alternatively spliced exons, we subjected the 49 genes to pathway analysis. As a result, we identified the EGFR/mTOR signaling pathway as the most significantly linked network by these alternative exons (Figures 1 and 3). There were seven alternative splicing events in this pathway. For example, the alternative exon of *Egfr* (epidermal growth factor receptor) suggested that the extracellular protein isoform was expressed via alternative polyadenylation located in an adjacent upstream exon that encoded the transmembrane domain [35]. Although the expected extracellular isoform of Egfr is interesting, the distinct functions of this isoform are unclear. The alternative exon of *mTor* (mammalian target of rapamycin) is part of the N-terminal predicted short protein isoform predictively, which has been reported to be regulated by an alternative splicing regulator, SAM68, during adipogenesis. It was also suggested to be involved in nonsense mediated mRNA decay [36]. Meanwhile, Tsc2 (tuberous sclerosis 2) was in the original 262 candidate exons but was eliminated from the 49 exons (Figure 3) [12] because the region affected by the alternative exon did not encode a functional domain.

Generally, differentially expressed genes affect biological phenomena. Thus, we also checked and extracted the differentially expressed genes associated with the EGFR/mTOR pathway, and assessed with their increased or decreased expression (Figure 3) [12]. Additionally, it was reported that protein expression of p53 could increase during the neuronal differentiation of P19 cells [37]. Including gene expressions and exons expression, various transcripts were observed to have altered in this pathway. However, it is speculated that upregulation of *Egfr* gene expression increases the potency of its signal, while increased expression of its alternative exon interferes with the signal via its imperfect extracellular isoform.

### Validations of gene and exon expressions in EGFR/mTOR pathway

**Figure 4:**
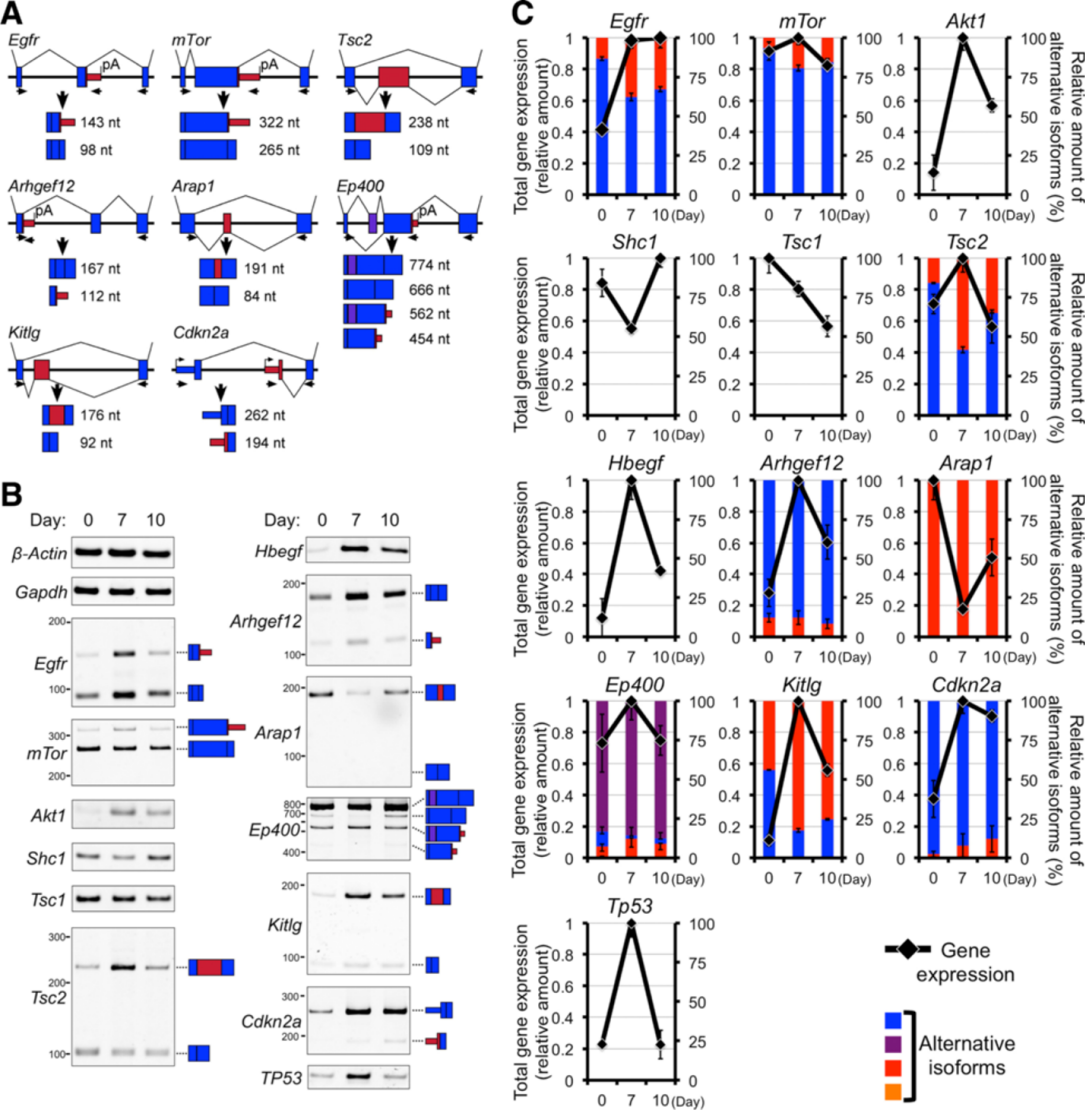
**Validation of exon and gene expressions in the EGFR/mTOR pathway. (A)** Schematic representation of alternative splicing in this pathway. Exons indicated by probeset seqs are shown in red and additional exons that predict complex alternative splicing are shown in violet. The mountain lines indicate the splicing patterns. **(B)** Gel images showing semi-quantitative RT-PCR performed using undifferentiated P19 cells (Day 0), P19 cells at the neuronal cell stage (Day 7), and P19 cells at the early glial cell stage (Day 10). Each isoform of a transcript is indicated on the right side. **(C)** Graphical representations of total gene expressions or the proportional changes of alternatively spliced products are shown on the right axis using black lines or on the left axis using colored columns, respectively. After densitometric analyses, the highest amount of total gene expression among three time points was considered as 1.0. Relative amounts of isoforms in total transcripts at each time point were considered as 100%. Error bars indicate the standard error.

Speculations based on gene expressions or exon expressions may contradict each other. We suspected that some comprehensive deposited data include partial or uncertain information; we confirmed experimentally the gene and exon expressions during neuronal differentiation of P19 cells. First, we examined the expression of alternatively spliced exons of *Egfr* using semi-quantitative RT-PCR. The proportions of the transcript that encodes extracellular isoform and full-length products changed from about 1.5:8.5 to 4:6 during neuronal differentiation of P19 cells (Figure 4). This was suggested by the splicing index value (SI) in our previous study [12]. The loss of domain type, such as an extracellular isoform, increased; therefore, we marked EGFR the inner red color on Figure 3. There is no experimental evidence of extracellular isoform’s functionality compared with the full-length product; therefore, it is difficult to determine the functional changes induced in these isoforms. In addition, the amounts of extracellular and full-length type transcripts increased by approximately 6.8-fold and 1.7-fold, respectively (Figure 4). This change also correlated with the change that was suggested by fold change (FC) in neuronal gene expression in a previous comprehensive analysis [12]. We indicated increased gene-expression of EGFR with the outer blue color on Figure 3.

In the case of another key factor in this network, the SI value suggested that the N-terminal short isoform-encoding transcript of *mTor* increased during neuronal differentiation, and the FC value suggested that expression of the gene did not increase [12]. Actually, the relative amounts of N-terminal isoform products did increase and the amount of total transcripts from the *mTor* gene did not remarkably change during differentiation (Figure 4). We indicated the increased loss of domain type, such as N-terminal short isoform of mTor, with the inner red color in Figure 3. Although the function of the mTor N-terminal isoform is unclear during differentiation, its transcript is degraded during adipogenesis [36]. Perhaps a truncated isoform that loses a functional protein domain loses its function. As mentioned above, mTOR signaling promotes cell proliferation [24, 38], and the N-terminal isoform lacks certain domains present in the full-length protein. Cell differentiation and cell proliferation are generally contrary phenomena. Therefore, this N-terminal alternative spliced isoform of mTor may be important for the neuronal differentiation of P19 cells.

Although extracellular isoform of Egfr may negatively affect this pathway, similar to the mTor N-terminal isoform, the full-length type *Egfr* transcripts increased. Therefore, we tested the expressions of genes and their exons that exist between EGFR and mTOR in the pathway and that were suggested to change by their SI and FC values. Typically, the *Akt1* (RAC-alpha serine/threonine-protein kinase) transcript increased 7-fold during neuronal differentiation (Figure 4). The change of *Akt1* alternative isoforms was not suggested by its SI value. The decrease of gene expression of *Tsc1* was suggested by its FC and was observed to change slightly during differentiation (Figure 4). Although the suggested change of *Tsc2* was validated, the effect of its alternative splicing is unclear. Another suggested downstream gene, *Shc1* (SHC-transforming protein 1), was validated and decreased during differentiation (Figure 4). Thus, gene expressions of *Egfr* and *Akt1* increased, but the expressions of other genes in this pathway did not. Besides mTor or Tscs, various target proteins of Akt1 have been identified [reviewed in [39]. We speculated that other target proteins may be dramatically upregulated by enhanced Akt1 potency, according to their gene expressions. In the case of mTor, its alternative N-terminal product may slightly negatively modulate the enhanced input signals.

Meanwhile, some upstream genes of the EGFR pathway, such as *Hbegf* (Heparin-binding EGF-like growth factor) and *Arhgef12* (Rho guanine nucleotide exchange factor 12) increased (Figures 3 and 4). The alternative isoform of *Arap1* (ArfGAP with RhoGAP domain, ankyrin repeat and PH domain 1) was not detected; however, the expression of its full-length product decreased during differentiation (Figure 4). In the case of *Ep400* (E1A binding protein p400), its expression increased and the alternatively spliced product may positively affect EGFR pathway according to its changed domain. Additionally, changes in indirectly related genes, such as Kitlg (KIT Ligand) and Cdkn2a (cyclin-dependent kinase inhibitor 2A) were also validated their gene- and exon- expressions during differentiation (Figures 3 and 4). However, the functional change caused by the domain modification by alternative splicing of Kitlg was unclear. It was difficult to determine the effects on EGFR signaling because upstream genes showed positive and negative changes.

### Time-course analysis of gene and exon expressions of Egfr

**Figure 5:**
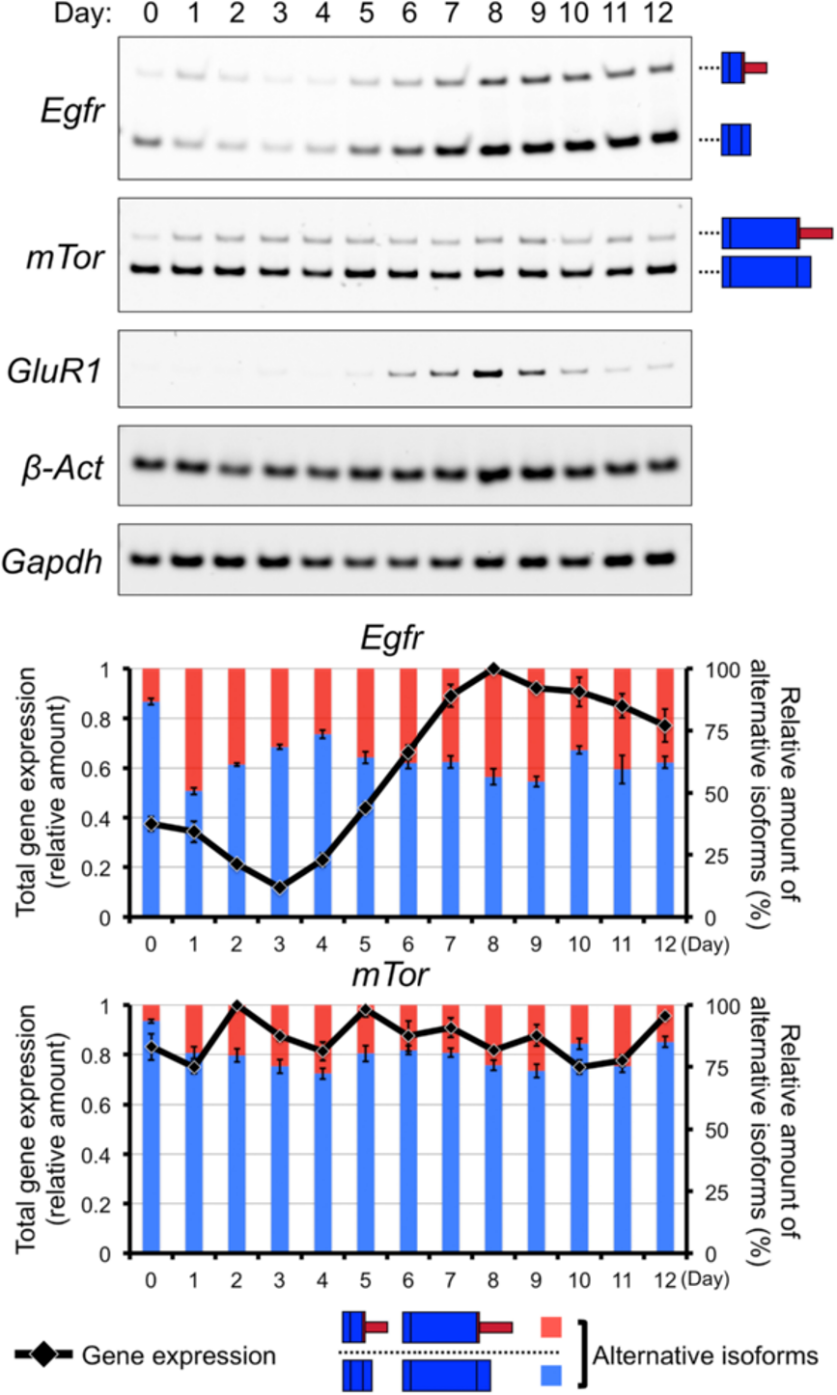
**Time-course experiments of alternatively spliced Egfr and mTor transcripts.** Detailed expression analyses of gene and exon expressions of *Egfr* and *mTor* are shown in the gel image. Using a series of cDNAs; from Day 0–12, exon expressions were examined. At the same time, gene expressions were quantified by the densitometric analyses of total transcripts. Graphical representations of gene or exon expressions are shown in the upper panel and lower panel, respectively. Error bars indicate the standard errors.

Autonomous regulation represents one possible explanation of the contradictory expressions of the *Egfr* gene and exons. For example, increased gene expression indirectly affects its alternative splicing to repress gene function. To test this hypothesis, we prepared a series of cDNAs from the undifferentiated stage to the glial cell stage of P19 cells. We then examined the most changed time points of *Egfr* gene or exon expression. The relative amount of the extracellular *Egfr* isoform increased in the day 1 sample during neural induction by retinoic acid (RA) under aggregation culture (Figure 5). Similar fast responses by alternative splicing have been observed in other genes during differentiation [40, 41]. The previously reported genes were also regulated by RA and/or cell aggregation. At this time point, the total amount of *Egfr* transcript showed little change (Figure 5). The proportion of the extracellular isoform then decreased, before rising again from day 4 to day 9: from neural induction to the neuronal cell stage of P19 cells (Figure 5). On the other hand, increased *Egfr* gene expression was observed from day 3 to day 8 (Figure 5). In other words, the elevation of *Egfr* gene expression was started 1 day before the proportional increase of its extracellular isoform and stopped 1 day before the isoform began to decrease again. Similarly, we tested *mTor* using a series of cDNAs. Its N-terminal isoform was elevated during the neural induction and differentiation stages, which was completely different from *Egfr*. Although the expression of the *Egfr* gene and its alternative isoforms expressions fitted with the autonomous regulation hypothesis and may rebalance their functions, we lack proof of the hypothesis. This should be addressed in our future work.

## Conclusions

Changes in alternative exons affect protein functions via alterations of amino acid sequences and functional domains. Our method extracted alterations of functional domains and identified key alternative splicing events. We found that the EGFR/mTOR signaling pathway was the most affected pathway. The importance of mTOR in neuronal differentiation has been reported, suggesting that this *in silico* extraction of alternative splicing network is a useful strategy. This strategy for the analysis of alternative splicing should be automated. The experimental validations of exon and gene expression by RT-PCR suggested that the increase in the products of genes such as *Egfr* and *Akt1* might increase the signal through the pathway during neuronal differentiation of P19 cells, meanwhile the alternative splicing events in *mTor* might control this pathway repressively. We speculate that our method will contribute to future studies of new molecular networks of alternative splicing regulation.

## Authors’ information

SA and HTTP are graduate students, and MT, TT and HS are faculties in Japan Advanced Institute of Science and Technology. The corresponding author is HS, who also belong to Center for Nano Materials and Technology, Japan Advanced Institute of Science and Technology. MO was a summer-time internship student in Japan Advanced Institute of Science and Technology and belongs to Department of Chemicals and Engineering, Miyakonojo National College of Technology. KK is a president of World Fusion Co., Ltd.

## Electronic supplementary material

Additional file 1: Table S1: List of primer sequences and PCR conditions. (XLSX 51 KB)

Additional file 2: Table S2: Results of the analyses in 262 DAS exons. Using filter conditions of the last column, 49 gene with 54 probesets are listed. (XLSX 255 KB)

## Abbreviations

P19 cells: P19 mouse embryonic carcinoma cells
DAS: Differentially alternatively spliced
UTR: Untranslated region
GO: Gene Ontology
EGFR: Epidermal growth factor receptor
mTOR: Mammalian target of rapamycin
SI: Splicing index
FC: Fold change
AKT1: RAC-alpha serine/threonine-protein kinase
SHC1: SHC-transforming protein 1
HBEGF: Heparin-binding EGF-like growth factor
ARHGEF12: Rho guanine nucleotide exchange factor 12
KITLG: KIT Ligand
CDKN2A: Cyclin-dependent kinase inhibitor 2A
ARAP1: ArfGAP with RhoGAP domain ankyrin repeat and PH domain 1
EP400: E1A binding protein p400.
